# Timing of Decompressive Craniectomy for Ischemic Stroke and Traumatic Brain Injury: A Review

**DOI:** 10.3389/fneur.2019.00011

**Published:** 2019-01-25

**Authors:** Aatman Shah, Saleh Almenawer, Gregory Hawryluk

**Affiliations:** ^1^Department of Neurosurgery, University of Utah School of Medicine, Salt Lake City, UT, United States; ^2^Division of Neurosurgery, Hamilton Health Sciences and McMaster University, Hamilton, ON, Canada

**Keywords:** TBI, stroke, decompressive hemicraniectomy, timing, herniation

## Abstract

While studies have demonstrated that decompressive craniectomy after stroke or TBI improves mortality, there is much controversy regarding when decompressive craniectomy is optimally performed. The goal of this paper is to synthesize the data regarding timing of craniectomy for malignant stroke and traumatic brain injury (TBI) based on studied time windows and clinical correlates of herniation. In stroke patients, evidence supports that early decompression performed within 24 h or before clinical signs of herniation may improve overall mortality and functional outcomes. In adult TBI patients, published results demonstrate that early decompressive craniectomy within 24 h of injury may reduce mortality and improve functional outcomes when compared to late decompressive craniectomy. In contrast to the stroke data, preliminary TBI data have demonstrated that decompressive craniectomy after radiographic signs of herniation may still lead to improved functional outcomes compared to medical management. In pediatric TBI patients, there is also evidence for better functional outcomes when treated with decompressive craniectomy, regardless of timing. More high quality data are needed, particularly that which incorporates a broader set of metrics into decision-making surrounding cranial decompression. In particular, advanced neuromonitoring and imaging technologies may be useful adjuncts in determining the optimal time for decompression in appropriate patients.

## Introduction

Decompressive craniectomy has been used to treat elevated intracranial pressure (ICP) resulting from various etiologies, especially ischemic and traumatic brain injuries. Given the inflexible confines of the skull, brain swelling from stroke or TBI can result in a compartment syndrome, increasing intracranial pressure (ICP). This can reduce cerebral perfusion pressure (CPP), cerebral blood flow (CBF), and oxygenation (1). If not acted upon, this can lead to brain ischemia, infarction, herniation, and death. There are various management strategies to treat elevated ICPs which include sedation, hyperventilation, hyperosmolar therapy, paralysis, hypothermia, barbiturates, and cerebrospinal fluid drainage (2). Decompressive craniectomy is a treatment option generally reserved for ICP elevation refractory to less invasive treatments (3).

Although decompressive craniectomy has been shown to effectively reduce ICP (4), there remains much controversy regarding its effect on overall clinical outcome, especially following TBI (5). Additionally, it is becoming clear that factors such as timing of decompressive craniectomy may play a significant role in determining the therapeutic benefit of this procedure. There are various studies providing insight into the optimal timing of decompressive craniectomy for victims of TBI and ischemic stroke and it is important for neurosurgeons to be aware of this data. The goal of this paper is to synthesize published findings regarding optimal timing for craniectomy for both malignant stroke and TBI in relation to time from injury and in relation to cerebral herniation.

## Timing of Craniectomy after Ischemic Stroke

### Decompressive Craniectomy for Stroke in the Animal Model

Some animal data have suggested that early decompressive craniectomy could yield better functional outcomes than late decompression or non-operative management. In two animal studies with standardized experimental conditions, rats undergoing decompressive craniectomy after MCA infarction had a significantly better outcome and had a reduction in infarct size when compared to the non-surgical groups (6, 7). These studies were based on the hypothesis that avoiding herniation and mesencephalic ischemia would improve prognosis. Doerfler et al. concluded that the decompressive craniectomy groups demonstrated better mortality and neurologic outcome when compared to the non-surgical group (6). It was further concluded that the animals treated with very early decompression (within 4 h) demonstrated significantly better neurologic outcomes and smaller infarct size compared to animals treated with later decompression. Forsting et al. also found that decompressive craniectomy improved outcomes, mortality, and infarct size when compared to the non-surgical group regardless of when the decompression occurred (7).

### Decompressive Craniectomy Within 48 h of Ischemic Stroke

Decompressive craniectomy following acute ischemic stroke has been studied in three relatively recent human randomized controlled trials. These studies analyzed the effects of decompressive surgery on mortality and functional outcome after malignant hemispheric stroke. The subsequent pooling and meta-analysis of these studies has generated very important insights as will be described.

DECIMAL (Decompressive Craniectomy in Malignant Middle Cerebral Artery Infarcts) was published in 2007. It assigned 38 patients to undergo surgery or medical management within 30 h of their initial stroke (8) (see Table 1). When compared to the medical therapy cohort, the cohort that underwent decompressive craniectomy demonstrated a mortality rate that was more than halved, and a 50% increase in the proportion of patients with only moderate disability.

**Table 1:** Decompressive craniectomy for stroke studies.

	**Author**	**Study design**	**Patients**	**Selection criteria**	**Treatment**	**Total no of patients**	**Time to DC**	**Mortality *n*(%)**	**Functional outcome at 6 months**	**Functional outcome at 12 months**	**Conclusions**
Stroke	Vahedi et al. (8)	Randomized controlled trial	Adult patients with MCA infarction	Patient age 18–55 years, within 24 h of a malignant MCA infarction, NIHSS ≥ 16,; >50% of the MCA territory involved on CT; DWI infarct volume >145 cm^3^	DC	20	Avg 20.5 ± 8.3 h (range, 7–43 h)	5 (25)	mRS score ≤ 3: 25%	mRS score ≤ 3: 50%	When compared to medical management, the DC group demonstrated an increase in the number of patients with moderate disability by more than half and demonstrated a reduction in the mortality rate by more than half.
					Medical management	18	NA	14 (78)	mRS score ≤ 3: 5.6%	mRS score ≤ 3: 22.2%
	Juttler et al. (9)	Randomized controlled trial	Adult patients with MCA infarction	Patient age 18–60 years, at least 2/3 of MCA territory infarction with basal ganglia involvement, NIHSS >18 for non-dominant hemisphere, NIHSS > 16 for dominant hemisphere, symptoms > 12 h but <36 h before possible DC	DC	17	Within 36 h after stroke	2 (11.8)	mRS score ≤ 3: 47%	mRS score ≤ 3: 47%	DC reduces mortality in large hemispheric stroke. Functional outcomes at 6 and 12 months were comparable between both groups
					Medical management	15	NA	8 (53.3)	mRS score ≤ 3: 27%	mRS score ≤ 3: 27%
	Hofmeijer et al. (10)	Randomized controlled trial	Adult patients with MCA infarction	Patient age 18–60, at least 2/3 of MCA territory stroke within 96 h of treatment, NIHSS score >16 right sided lesions or >21 left sided lesions	DC	32	Within 96 h after stroke	7 (22)	NA	mRS score ≤ 3:25%	DC can improve fatality and functional outcomes when performed within 48 h; however, when delayed up to 96 h, there was no improvement in functional outcomes.
					Medical management	32		19(59)	NA	mRS score ≤ 3:25%
	Vibbert et al. (3)	Randomized controlled trial	Adult patients with MCA infarction	Patient age 18–60, at least 2/3 of MCA territory stroke within 96 h of treatment, NIHSS score >16 right sided lesions or >21 left sided lesions	DC	32	Within 96 h after stroke	NA	NA	mRS score ≤ 3:25%	DC can improve fatality (absolute risk reduction of 38%); however, there was no improvement in functional outcomes.
					Medical management	32		NA	NA	mRS score ≤ 3:25%
	Schwab et al. (12)	Prospective cohort	Adult patients with MCA infarction	Patients younger than 70, >50% MCA territory infarction noted on CT imaging	Early DC	31	Within 24 h after stroke	5 (16)	Avg Barthel Index Score: 68.8	NA	Earlier DC was associated with lower mortality. There was a trend toward better functional outcomes, and the patients spent less time in the ICU
					Late DC	32	>24 h after stroke	11 (34.4)	Avg Barthel Index Score: 62.6	NA
					Medical management	55		43 (78)	Avg Barthel Index Score: 60	NA
	Wang et al. (13)	Retrospective cohort	Adult patients with MCA infarction	Patients with 1st stroke >90% MCA infarction	Early DC	11	Within 24 h after stroke	3 (27)	Mean Glasgow Outcome Score: 2.5	NA	While the mortality rates were comparable between groups, severe disability may be reduced in early treated patients
					Late DC	10	>24 h after stroke	3 (30)	Mean Glasgow Outcome Score: 2.45	NA
					Medical management	41		9 (22)	Mean Glasgow Outcome Score: 2.73	NA
	Cho et al. (14)	Retrospective cohort	Adult patients with MCA infarction	Patients with > 50% MCA infarction with NIHSS score > 20	Ultra-early DC	12	Within 6 h after stroke	1 (8.3)	Avg Barthel Index Score: 70	NA	DC before neurologic compromise may reduce the mortality rate and increase the conscious recovery rate
					Delayed DC	30	>6 h after stroke	11 (36.7)	Avg Barthel Index Score: 52.9	NA
					Medical management	10		8 (80)	Avg Barthel Index Score: 55	NA
	Mori et al. (15)	Retrospective cohort	Adult patients with MCA infarction	Patients <85 years of age with patients with embolic hemispheric infarction volume > than 200 cm3	Early DC	21	DC before brain herniation	4 (19.1)	Avg Barthel Index Score: 52.9	NA	Early DC before the onset of brain herniation should be performed to improve mortality and functional recovery. DC after signs of herniation may be too late for functional benefit
					Late DC	29	DC after brain herniation	8 (27.6)	Avg Barthel Index Score: 26.9	NA
					Medical management	21		15 (71.4)	Avg Barthel Index Score: 28.3	NA
	Elsawaf et al. (16)	Prospective cohort	Adult patients with MCA infarction	Patients with malignant MCA infarction	DC based on clinical status	27	DC with deterioration of consciousness	14 (52)	Mean mRS Score: 4.7	NA	Early prophylactic DC yields better clinical and radiographic outcomes than DC based on clinical status
					Early DC	19	DC within 6 h of stroke	2 (10.5)	Mean mRS Score: 3.5	NA

DESTINY (Decompressive Surgery for the Treatment of Malignant Infarction of the Middle Cerebral Artery) was also published in 2007. It enrolled 32 patients within 36 h of stroke (9). This randomized study demonstrated that craniectomy reduces mortality in large hemispheric stroke. Like DECIMAL, this study demonstrated a reduction in death rates in the surgical cohort, but also like DECIMAL the sample size of the DESTINY trial was not sufficient to draw conclusions regarding functional outcome.

Because the above studies were underpowered to assess differences in functional outcomes, the HAMLET (Hemicraniectomy After MCA Infarction With Life Threatening Edema Trial) trial was initiated. This third RCT was published in 2009 and was crucial in adding to the body of literature regarding decompressive surgery following acute ischemic stroke (10). HAMLET enrolled 64 patients up to 96 h after stroke. This RCT demonstrated that when decompressive craniectomy is delayed up to 96 h, there was no improvement in functional outcomes in survivors. The percentage of patients with a mRS score less than or equal to three at 1 year follow up were comparable between the decompressive craniectomy and control group (25% in both groups). It should be noted that three patients in the surgical group and three patients in the medical group had a fixed and dilated pupil on enrollment which means that roughly 20% of the study population demonstrated signs of herniation prior to treatment. Because 20% of this study population had already demonstrated signs of herniation, it can be argued that delayed craniectomy may be too late to impart any functional benefit.

A meta-analysis of the three studies was performed by Vahedi et al. on the patients treated with surgery within 48 h in the DECIMAL and DESTINY trials as well as the first 23 patients of the HAMLET trial (11). In this meta-analysis of 93 patients crossover was minimal: there was only one crossover from non-operative treatment to decompressive surgery included in this analysis from the DESTINY trial. The results demonstrated increased favorable functional outcome compared to the medical cohort (11). In this paper, 43% of the decompressive craniectomy group had a modified Rankin scale (mRS) score of 0–3 compared to 21% in the control group. It should be noted that from the human studies presented thus far, there have been no direct comparisons between outcomes of early vs. late decompressive craniectomy.

The findings from the meta-analysis performed by Vahedi et al. was further corroborated by the findings of Vibbert et al. This study contained 64 patients with acute ischemic stroke in the MCA territory who presented within 96 h of symptom onset. The patients were randomized to receive medical management or surgical intervention (3). The primary outcome was the modified Rankin scale (mRS) at 12 months which was stratified as good outcome (0–3) and severe disability or death (4–6). Twenty-four out of 32 patients in each arm had a mRS score >3 at 12 months, and rates of severe disability were also similar between groups. The risk of death was significantly reduced in the surgical group (absolute risk reduction of 38%; *P* = 0.002). The authors performed subgroup analyses of patients who underwent surgery in <48 h and patients who underwent surgery after 48 h. For patients who underwent surgery within 48 h of stroke, the risk of death and an mRS score >4 were reduced (respectively: ARR, 59%; 95% CI, 33–84; ARR 30%; 95% CI, 1–59) (3).

Vibbert et al. then performed an updated meta-analysis with their cohort of patients and patients from the aforementioned DECIMAL, DESTINY, and HAMLET trials who underwent decompressive surgery within 48 h (3). Corroborating their prior findings, the data suggest a significant reduction in risk of death (ARR, 49.9%; 95% CI, 33.9–65.9) and in the risk of severe disability at 12-months (ARR, 41.9%; 95% CI, 25.2–58.6). While not statistically significant, there was a notable trend toward reduction in risk of poor outcome (12 month mRS score >3—ARR, 16.3%; 95% CI, −0.1–33.1).

In summary, decompressive craniectomy within 96 h of malignant MCA stroke did not reduce poor outcomes at 1 year; however, there seems to be a trend toward reduction in death and moderate-to-severe disability (mRS score>4) when surgery was performed within 48 h from the stroke. It is possible that a significant portion of patients 96 h from an acute ischemic stroke may already exhibit herniation, and decompressive craniectomy at this time may be too late to impart functional benefit. Unfortunately, analysis of outcome in relation to herniation events was insufficiently described in these studies, and it is not possible to determine whether early surgery is beneficial due to avoiding herniation or whether there is a benefit to early surgery independent of herniation. The benefit of early surgery in the cohort by Vibbert et al. was not as great in magnitude as in HAMLET; however, this may have been due to a longer interval between symptom onset and surgical treatment in HAMLET (mean 31 h) than in DECIMAL (mean 16 h) and DESTINY (mean 24 h).

### Decompressive Craniectomy Within 24 h of Ischemic Stroke

Other data have suggested that early decompressive craniectomy within 24 h of stroke could yield even better functional outcomes. Schwab et al. conducted a prospective observational trial where the patient population was stratified by early craniectomy (<24 h after symptom onset) and late craniectomy (>24 h), with additional comparison to a natural history group (12). Patients were included in the study if they had >50% MCA territory infarction noted on CT imaging. The mean time between symptoms and surgery was 21 h (range, 8–42 h) in the early craniectomy group and 39 h (range, 6–112 h) in the late group. This difference approached statistical significance (*p* = 0.07). Mortality was 16% (5/31) in the early group, 34.4% (11/32) in the late group, and 78% (43/55) in the historical controls (12). The late group demonstrated uncal herniation in 24 of 32 patients (75%) whereas only 4 of 31 patients (13%) in the early group demonstrated uncal herniation. Length of stay in the ICU was 7.4 days for the early group and 13.3 days in the late treated group (*p* = 0.05). Functional outcome measured by the Barthel Index (BI) demonstrated a higher mean score for the early group with an average score of 70 vs. 62.6 in the late group. There was a trend toward better outcomes with early craniectomy, however, the data were not statistically significant. Overall, this study demonstrated that early craniectomy was an efficacious approach for treating malignant MCA infarction when the patients were treated before signs of herniation. The mortality rate was lower, there was a trend to better functional outcome, and the patients spent less time in the ICU.

The data presented by Schwab et al. were further corroborated by smaller series published by Wang et al. and Cho et al. In a retrospective study of 21 patients, Wang et al. compared the outcomes of early decompression (<24 h) to late decompression (>24 h) (13). While the mortality rate was comparable, Wang et al. demonstrated that severe disability may be reduced in early treated patients. Cho et al. further corroborated this data, and demonstrated the positive results in association with ultra-early decompression defined as decompression within 6 h of symptom presentation (14). The Cho et al. study reported only a cohort of 52 patients and demonstrated that the acute mortality rate was statistically lower for the ultra-early group (8.3%) compared to the delayed decompression group (>6 h) and the no surgery group (36.7 and 80%, respectively, all *p*-values < 0.001). The ultra-early group also had better prognosis for conscious recovery (91.7%) compared to the delayed decompression group and the no surgery group (55 and 0%, respectively). While more data are needed, the published data give credence to the idea that early craniectomy performed within 24 h yields better mortality and functional outcomes. Moreover, this study suggests that the benefit to early surgery may not merely stem from an avoidance of herniation.

### Decompressive Craniectomy for Ischemic Stroke Based on Clinical Correlates of Herniation

While the previous studies demonstrated benefit from early decompression, a key limitation was insufficient delineation of the role of herniation events in distinction to merely performing early surgery. Indeed, there have been more recent studies that indicate that the timing of craniectomy should be based on clinical features rather than on a strict temporal scale given the variations in when herniation events occur in the clinical course of different patients. A retrospective study by Mori et al. analyzed the outcomes of 71 patients with embolic hemispheric infarctions (infarct volume >200 cm^3^) who were stratified into 3 groups: non-operative management, decompressive craniectomy after brain herniation (late surgery group), and decompressive craniectomy before brain herniation (early surgery group) (15). This study utilized the Glasgow Coma Scale (GCS), changes in mental status, and anisocoria as clinical indicators for herniation. The mortality at 1 and 6 months in the non-operative group was 61.9 and 71.4%, respectively. The mortality at 1 and 6 months in the late surgery group was 17.2 and 27.6%, respectively, (*p* = 0.01) and was even better in the early surgery group −4.8 and 19.1%, respectively. The Glasgow Outcome Scale (GOS) and Barthel Index (BI) were employed as functional outcome measures at 6 months. The GOS scores of the early surgery group were better than those of the late surgery group (*p* = 0.05). The average BI score of the early surgery group (52.9 ± 34.2) were improved from those of the late surgery group (26.9 ± 30.4) (*p* = 0.05). The late surgery group had a comparable BI score to the non-operative group (28.3 ± 42.2), which indicates that surgery after signs of herniation may be too late to yield functional benefit. Mori et al. thus concluded that an effort should be made to perform early decompressive craniectomy before the onset of brain herniation in patients with malignant cerebral infarction. Mori et al. also concluded that embolic stroke patients with >200 cm^3^ volume of infarction and shift of the midline structures on a follow-up CT 2 days after ictus are more likely to herniate and would benefit from decompressive craniectomy.

Mori et al. advanced the field by conceptualizing outcome in relation to clinical indicators of herniation. With this in mind, Elsawaf et al. published a recent and important prospective study comparing outcomes of early decompression (within 6 h of presentation) and decompression based on clinical features of deterioration. Forty-six patients with large hemispheric MCA infarction were divided randomly into two groups: Group I in which patients were followed until deterioration of level of consciousness, and Group II in which patients were operated within 6 h of presentation regardless of clinical signs of deterioration or radiographic features (16). While both groups demonstrated improvement in conscious level, motor power, and functional outcome, there was significant improvement (*p* < 0.05) in functional outcome in group II based on the mRS. Group I demonstrated increased progression of infarct volume when compared to Group II, and also had a morality of 52% due to delay in surgery compared to 10% in Group II. This study found better clinical and radiographic outcomes for patients with large hemispheric MCA infarction who were operated on prophylactically within 6 h of infarction without waiting for deterioration of level of consciousness.

While there are concerns that very early decompression surgery might potentially be unnecessary, the presented data demonstrate that decompression after the onset of herniation symptoms is less effective, or may even be ineffective in reducing mortality and improving neurological outcome. While more data are required, current studies suggest that stroke patients with malignant infarction >200 cm^3^ and follow up CT at 2 days from symptom onset which demonstrate shift of the midline structures are likely to herniate and would benefit from early decompressive craniectomy.

## Timing of Craniectomy after Traumatic Brain Injury

### Decompressive Craniectomy for Traumatic Brain Injury (TBI) in the Animal Model

Preliminary data from TBI animal models treated with decompressive craniectomy have suggested that decompressive craniectomy could reduce edema formation and prevent secondary expansion of the original contusion when compared to non-operative management. Zweckberger et al. utilized a controlled cortical impact model of TBI in a cohort of mice to study the influence of decompressive craniectomy on secondary contusion expansion and brain edema formation, and to determine optimal timing of decompressive craniectomy (25). It was determined that in the surgical groups, there was no secondary expansion of the original contusion and there was a 52% reduction of brain edema compared to the non-surgical group. These benefits were seen with decompressive craniectomy when performed up to 3 h after the initial trauma. Tomura et al utilized a fluid percussion injury model of TBI in a cohort of rats to investigate the influence of decompressive craniectomy on post traumatic brain edema formation. It was found that the non-surgical group demonstrated less cortical water content and greater AQP4 expression when compared to the decompressive craniectomy group.

### Decompressive Craniectomy More Than 24 h After TBI

Although there is some controversy regarding the use of decompressive craniectomy in ischemic stroke patients, the use of decompressive craniectomy following human TBI has certainly been more controversial. Three RCTs have analyzed the outcomes of TBI patients after late decompressive craniectomy (more than 24 h from the injury) (see Table 2). The DECRA (Decompressive Craniectomy in Diffuse Traumatic Brain Injury) trial published by Cooper et al. in 2011 was a landmark RCT which informed the outcomes of TBI patients with diffuse injuries who were treated with decompressive craniectomy within 72 h of injury (17). In this study, 155 patients with refractory ICPs >20 mmHg for 15 min within a 1-h period were randomized into a decompressive craniectomy group (bifrontal decompressive craniectomy) or a maximal medical management group. On average, the time from injury to surgery was 38.1 h, with a range of 27.1–55.0 h. In this study, Cooper et al. determined that bifrontal decompressive craniectomy decreases ICP and the length of stay in the intensive care unit, but is associated with more unfavorable outcomes. There are, however, some criticisms involving the DECRA trial. First, the randomization was uneven between the 2 groups. There were more patients with non-reactive pupils in the decompressive craniectomy group than the medical therapy group (27 vs. 12%, respectively [*p* = 0.04]). It can be argued that more patients in the decompressive craniectomy group already demonstrated signs of herniation prior to treatment which may obfuscate the therapeutic benefit from a decompressive craniectomy. Indeed, the harm associated with decompression was no longer statistically significant when a statistical control for unreactive pupils was performed. Other issues included the relatively small sample size and that only bifrontal decompressive craniectomy without falx sectioning was allowed. Some researchers believe DECRA was too aggressive and that ICP elevations should have been sustained for longer durations prior to considering surgery. Lastly, there were no standardized rehabilitation protocol for the enrolled patients.

**Table 2:** Decompressive craniectomy for TBI studies.

	**Author**	**Study design**	**Patients**		**Treatment**	**Total no of patients**	**Time to DC**	**Mortality *n*(%)**	**GOS at 6 months**	**GOS at 12 months**
TBI	Cooper et al. (17)	Randomized controlled trial	Adults with TBI	Age 15–59 years, severe, non-penetrating brain trauma	DC	73	Performed within 72 h after injury; a large bifrontotemporoparietal craniectomy with bilateral dural opening	14 (19)	Median = 3 (IQR 2–5)	NA	DC decreases ICP and the length of stay in the intensive care unit, but is associated with more unfavorable outcomes.
					Medical management	82	NA	15 (18)	Median = 4 (IQR 3–5)	NA
	Hutchinson et al. (18)	Randomized controlled trial	Adults with TBI	Age 10–65, abnormal CT scan of the brain, intracranial-pressure monitor already in place, and have raised intracranial pressure (>25 mm Hg for 1–12 h)	DC	202	Performed at any time. 44% were enrolled after 72 h	59 (30.4)	Favorable outcomes (upper severe disability or better): 42.8%	Favorable outcomes (upper severe disability or better): 45.4%	When compared to medical management, DC resulted in lower mortality and higher rates of vegetative state, lower severe disability, and upper severe disability. The rates of moderate disability and good recovery were comparable between both groups.
					Medical management	196	NA	93 (52)	Favorable outcomes (upper severe disability or better): 34.6%	Favorable outcomes (upper severe disability or better): 32.4%
	Qiu et al. (19)	Randomized controlled trial	Adults with TBI	Patient age 18–65, acute post-traumatic brain swelling on CT with > 5 mm midline shift, contusions <25 ml, compressed basal cisterns, and GCS 8 or less	Unilateral DC	37	DC for all patients within 2 to 24 h after admission	10 (27)	1: 10 (27%); 2: 1 (3%); 3: 5 (14%); 4: 6 (16%); 5: 15 (41%)	4 or 5 (56.8%)	Unilateral DC is superior to control temporoparietal craniectomy in lowering ICPs, reducing the mortality rate, and improving neurological outcomes.
					Control (unilateral routine temporoparietal craniectomy)	37	DC for all patients within 2 to 24 h after admission	21 (57)	1: 21 (57%); 2: 0 (0%); 3: 4 (11%); 4: 7 (19%); 5: 5 (14%)	4 or 5 (32.4%)
	Taylor et al. (20)	Randomized controlled trial	Pediatric patients with TBI		DC	13	DC was performed at a median of 19.2 h (range 7.3–29.3 h).	3 (23.1)	Favorable: 7 (53.8%); Unfavorable: 6 (46.2%)	NA	DC may be superior to medical management of in children with TBI in reducing ICP and improving functional outcome and quality of life.
	Cianchi et al. (21)	Retrospective cohort	Adults with TBI	186 patients with TBI were retrospectively studied from 2005–2009	Early DC	41	DC was performed within 24 h of TBI	12 (29.3)	Average GOS = 3.3	NA	Hospital mortality rates and Glasgow Outcome Scale at 6 month follow up were comparable between all groups
					Late DC	21	DC was performed after 24 h of TBI	6 (28.6)	Average GOS = 3.0	NA
					Medical management	124		30 (24.2)	Average GOS = 3.6	NA
	Bagheri et al. (22)	Prospective cohort	Adults with TBI	Severe TBI patients with midline shift > 5 mm and who were candidates for DC according to their initial brain CT scan from 2011–2014.	Early DC	61	DC performed 4.5 ± 2 h after trauma	NA	GOS > 3, 54.1% (33 patients)	NA	Patients whose age was >60 and a GCS <5 did not benefit from early decompressive craniectomy
	Jagannathan et al. (23)	Retrospective cohort	Pediatric patients with TBI	23 patients age < 18 who underwent DC for Trauma were analyzed 1995–2006	DC	23	DC performed on avg 68 h (range 24–192)	7 (30.4)	NA	Avg GOS at 2 years = 4.2 (median 5)	Although the mortality rate remains high, DC is effective in reducing ICP and is associated with good outcomes in survivors (81% returning to school)
	Shackelford et al. (24)	Retrospective cohort	Adults with TBI	Patients with combat-related brain injury between 2005 and 2015 who underwent DC at deployed surgical facilities	DC	486	Quintile 1: DC 30–152 min after TBI; Quintile 2: DC 154–210 min after TBI; Quintile 3 DC 212–320 min after TBI; Quintile 4: DC 325–639 min after TBI; Quintile 5: DC 665–3,885 min after TBI	Quintile 1: 23; Quintile 2:7%; Quintile 3: 7%; Quintile 4: 19%; Quintile 5: 14%	NA	NA	Mortality was significantly lowered when time to craniectomy occurred within 5.33 h of injury

After the DECRA trial, the therapeutic effect of decompressive craniectomy in TBI patients remained unclear, particularly in patients with focal pathology and when a lateral decompression is performed. In 2016, Hutchinson el al. published a multicenter (48 centers, 19 countries) RCT study named RESCUEicp (Trial of Decompressive Craniectomy for Traumatic Intracranial Hypertension) in which a cohort of 408 patients with TBI and refractory elevated ICP (>25 mmHg for at least 1 h) were randomized into a decompressive craniectomy group or a maximal medical therapy group (18). In this pragmatic study, 44% of patients were enrolled after 72 h. RESCUEicp was intended to study a distinct population of patients as compared with DECRA. The DECRA trial looked at decompression within 72 h after diffuse TBI, whereas the RESCUEicp trial analyzed decompressive craniectomy as salvage therapy for refractory intracranial hypertension. Moreover, patients with intracranial hematoma were not included in DECRA trial, but accounted for about 20% of the RESCUEicp trial. Unilateral craniectomy was not permitted in DECRA trial but was allowed in the RESCUEicp trial. At 6 months, the patients in RESCUEicp's decompressive craniectomy group exhibited lower mortality but higher rates of vegetative state, “lower severe” disability, “upper severe” disability, and comparable rates of moderate disability and “good recovery” when compared to the medical management group. It should also be noted that in the subgroup analysis comparing decompressive craniectomy performed before 72 h and at 72 h or more, there were no differences noted in functional outcomes. In interpreting this trial it is important to consider that 37.2% (73 patients) of the patients in the medical group ultimately underwent decompressive craniectomy. Notably, ten patients were excluded from analysis due withdrawal/lack of valid consent. Seven additional patients in the medical group were lost to follow-up. It is particularly important to consider that the majority of the patients in the RESCUEicp trial had diffuse injuries (78.6% of all study patients between the surgical and medical therapy groups) and underwent bifrontal decompressions (81.3% of the surgical group) despite the intent to enroll a distinct population from DECRA. With this in mind, the authors of this manuscript view RESCUEicp as confirming the findings of DECRA without substantially informing the use of decompressive craniectomy in patients with focal pathology, and the role of lateral decompressions.

Due to the paucity of data analyzing the importance of timing of decompressive craniectomy in outcomes of TBI patients, a meta-analysis published by Zhang et al. demonstrated that early decompressive craniectomy within 36 h could result in better prognosis based on the Glasgow Outcome Scale scores at 6 months when compared to patients operated on >36 h from the initial injury (5). The meta-analysis included 10 studies with four randomized controlled trials. On sub-group analysis, Zhang et al. determined that decompressive craniectomy could reduce mortality rate, lower ICPs, decrease ICU stay, but could also increase complication rate.

### Decompressive Craniectomy Within 24 h of TBI

While the aforementioned studies analyzed outcomes following decompressive craniectomy performed more than 24 h from time of injury, there have been efforts to analyze outcomes in TBI patients treated with early decompressive craniectomy within 24 h of injury. To that end, Cianchi et al. published their findings from a retrospective analysis which looked at the outcomes of early vs. late decompressive craniectomy compared to maximal medical management in treating TBI patients (21). In this study, 186 TBI patients were divided into early decompressive craniectomy (surgery within 24 h of TBI), late decompressive craniectomy (surgery after 24 h, on average 7.7 days after TBI), and maximal medical management groups. Hospital mortality rates and Glasgow Outcome Scale at 6 month follow up were comparable between all groups; however, the 6 month mortality rate was significantly less for the maximal medical management group compared to the early and late decompressive craniectomy groups (29, 48.8, 42.9%, respectively [*p* = 0.02]) (21). One of the main limitations of this analysis is the retrospective study design. Inherently, patients in the control group had intracranial pressures that were adequately treated with medical therapy whereas patients who received decompressive craniectomy failed medical therapy. It is therefore reasonable to conclude that the patients who underwent decompressive craniectomy had, on average, a more severe TBI. A more appropriate control group would include patients who were non-responders to medical treatment who were not treated with late decompressive craniectomy; however, there are obvious ethical considerations limiting such a study design.

To better address the importance of early decompressive craniectomy in TBI patients, Qiu et al. published an RCT analyzing the outcomes of early decompressive craniectomy in TBI patients (19). Seventy-four patients were randomized to either unilateral decompressive craniectomy or a control group which consisted of a unilateral routine temporoparietal craniectomy. All surgery occurred between 2 and 24 h (average 5.8 h) after admission. Enrolled patients needed to demonstrate >5 mm of midline shift on CT and compression of the basal cisterns. In this RCT, the entire cohort had progressed to some form of radiographic herniation. The mortality rates at 1 month after treatment were 27% in the unilateral decompressive craniectomy group and 57% in the control group. At 1 year follow up, good neurological outcome (GOS Score of 4–5) were 56.8% for the decompressive craniectomy group and 32.4% for the control group (*p* = 0.035). In contrast to the previous stroke studies which demonstrated that decompressive craniectomy after herniation does not confer any functional benefit, Qiu et al. concluded that unilateral decompressive craniectomy after radiographic signs of herniation may be superior to the control group at lowering ICPs, reducing the mortality rate, and improving functional outcome. It should be reiterated that all surgeries were performed within 24 h which is considered to be “early” compared to the timing of decompression reported in most of the TBI in the literature.

Bagheri et al. corroborated the findings of Qiu et al. and published their findings from a prospective study of 61 patients who underwent rapid decompressive craniectomy (within 4.5 ± 2 h) after trauma to assess factors associated with prognosis and outcome (22). Of the 61 patients, 54.1% demonstrated favorable functional outcomes; however, patients with ages older than 60 years, bilateral non-reactive mydriasis, critical head injury (GCS<5), or with >1 cm midline shift had worse outcomes. Bagheri argued that patients whose age was >60 and a GCS <5 did not benefit from early decompressive craniectomy.

Lastly, a large retrospective review involving 486 patients with combat related TBI who underwent decompressive craniectomy demonstrated that decompression within 5.33 h from TBI was associated with improved survival (24). The mortality of the patients were reported by time interval related quintiles: quintile 1 was defined as decompressive craniectomy 30–152 min after TBI, quintile 2 was defined as decompressive craniectomy 154–210 min after TBI, quintile 3 was defined as decompressive craniectomy 212–320 min after TBI, quintile 4 was defined as decompressive craniectomy 325–639 min after TBI, and quintile 5 was defined as decompressive craniectomy 665–3,885 min after TBI. The postoperative mortality was 23, 7, 7, 19, and 14% respectively. Mortality was significantly lowered when time to craniectomy occurred within 5.33 h of injury. While providing some insight into the possible importance of ultra-early decompressive craniectomy on survival, the retrospective design and the lack of long term functional outcome data limits the conclusions that can be drawn from this study.

Although more research is needed, decompressive craniectomy remains a frequently performed treatment—generally of last resort—for many patients with severe TBI. Much additional research is needed to optimize how and when this surgery is performed. In contrast to the findings in the stroke data, preliminary data for TBI studies demonstrate that decompressive craniectomy after acute herniation may still be beneficial in improving mortality and functional outcomes. Although more data are needed, TBI patients treated with early decompressive craniectomy seem to have lower mortality and potentially better functional outcomes than TBI patients treated with late decompressive craniectomy. As with the stroke data, the analysis of outcome for TBI patients in relation to herniation events was insufficiently described in relevant studies, and it is not possible to determine whether early surgery is beneficial due to avoiding herniation or whether there is a benefit to early surgery independent of herniation. While the larger RCTs indicate that decompressive craniectomy may increase the survival rate and concomitantly increase rates of severe disability including vegetative state, subsequent trials with a shorter duration to decompressive craniectomy have demonstrated improved functional outcomes and less mortality.

### Early Decompressive Craniectomy in Pediatric TBI Patients

Some published data demonstrate that early decompressive craniectomy may be beneficial in the pediatric population. To that end, Taylor et al. published the only RCT analyzing outcomes of early decompressive craniectomy in the pediatric population (20). Twenty-seven children who had sustained ICP elevation after TBI were randomized to the medical management group or the decompressive craniectomy group. Early bitemporal decompressive craniectomy was performed for the surgical group at a median of 19.2 h (range 7.3–29.3 h) from the time of TBI. Outcome was assessed 6 months after the TBI using a modification of the Glasgow Outcome Score (GOS) and the Health State Utility Index. At 6 months, 54% of children in the decompressive craniectomy group had good outcomes or mild disability at 6 months compared to 14% of children in the control group. Taylor et al. concluded that in pediatric TBI patients with refractory ICPs, patients treated with early decompressive craniectomy are more likely to have reduced ICPs and improved functional outcome than children treated with maximal medical therapy alone. While this is the only RCT published regarding decompressive craniectomy in the pediatric population, this study has received some criticism because it involved an unusual decompressive surgery in which the dura was not opened, and because it accrued a small number of patients over a long study period.

Jagannathan et al. corroborated the findings from Taylor et al. in their retrospective review on the outcomes of 23 pediatric patients who underwent decompressive craniectomy for TBI (23). The time to decompressive craniectomy was on average 68 h (range 24–192). Despite having longer time to decompressive craniectomy compared to Taylor et al., the mean GOS score at the 2-year follow-up examination was 4.2 (median 5). At latest follow up, 81% of the patients returned to school, and only 18% were dependent on caregivers. It should be noted that the outcomes in the Taylor et al. cohort were analyzed at 6 months, whereas the outcomes in the manuscript by Jagannathan et al. were analyzed at 2 year follow up, substantially confounding a comparison of the two trials. Although more data are needed, it is possible that earlier decompression may not be as important in improving long term outcomes in the pediatric population as has been shown in the adult population.

With the limited data at hand, it appears that the pediatric population has better functional outcomes with decompressive craniectomy regardless of timing when compared to medical management. Unfortunately, direct comparisons between early and late decompressive craniectomy have not been made in the pediatric population. Larger RCTs with direct comparisons will be needed to determine if timing plays a role improving outcomes in the pediatric population.

### Future Directions: Decompressive Craniectomy Based on Biologic and Radiographic Metrics

While there are data validating the benefits of early craniectomy based on specific time windows and clinical correlates of herniation, there are growing data that there may be other biologic and radiographic metrics to help guide timing of decompressive craniectomy for TBI and stroke. Strict control of intracranial pressures and cerebral perfusion pressures alone does not necessarily prevent cerebral hypoxia (26). Recent data have demonstrated that measurement of brain tissue oxygen tension (P_bt_O_2_) may more precisely measure the adequacy of cerebral perfusion, and could be a useful adjunct for deciding on the timing of decompressive craniectomy (27). A P_bt_O_2_ below 20 mmHg has been associated with poor outcomes in TBI patients (28). Reithmeier et al. published data on the effects of decompressive craniectomy on ICPs and P_bt_O_2_ based on the continuous monitoring of 15 patients and determined that P_bt_O_2_ monitoring could serve as a useful tool for timing craniectomy (2). One criticism of P_bt_O_2_ is that its measurements are based on data from the confines of a small volume of brain tissue which may not adequately reflect the oxygenation of a larger expanse of compromised brain. Other potentially useful biologic metrics include the pressure reactivity index (PRx) which is the correlation coefficient between mean intracranial pressure (ICP) and mean arterial blood pressure. This could be used as a surrogate marker of cerebrovascular impairment (29). There have also been preliminary data suggesting that surrogates for blood-brain-barrier disruption, defined by a ratio of total CSF protein concentrations to total plasma protein concentration, may also be useful for prognosis and treatment (30). Advances in imaging modalities may also be utilized to guide the treatment trajectory. The infarct growth rate (IGR) between two CT scans may also be a useful tool for timing craniectomy. Kamran et al. published a retrospective, multicenter cross-sectional study of 182 patients to identify factors for selecting the timing of craniectomy (31). The IGR on the second CT was one of the five factors identified as having the strongest association with craniectomy. Patients who survived without surgery had the slowest IGRs. On another retrospective cohort of 137 patients, Kamran et al. demonstrated that IGR was identified as an independent predictor of early surgery (32). The second infarct growth rate [IGR2] >7.5 ml/hr was associated with surgery under 48 h. Both first infarct growth rate [IGR1] and second infarct growth rate [IGR2] were nearly double in patients with early surgery within 48 h. Although more data are needed, monitoring the infarct growth rate could help determine when a neurosurgeon should pursue decompression. While promising, these biologic and radiographic metrics still require more data before they are used to counsel patients regarding treatment course and prognosis.

## Conclusion

Although there is much controversy surrounding optimal timing of decompressive craniectomy in patients with stroke and TBI, data have suggested that early decompression within 24 h has a tendency to improve mortality and functional outcomes for both conditions when compared to decompression performed after 24 h. In stroke patients, decompression before clinical signs of herniation yields improved functional outcomes when compared to decompression after clinical signs of herniation. Surgery after clinical deterioration may be too late to impart any functional benefit in stroke patients. In contrast to the stroke data, preliminary TBI data have demonstrated that decompressive craniectomy after signs of herniation may still lead to improved functional outcomes compared to medical management. In adult TBI patients, early decompressive craniectomy within 24 h may improve mortality and functional outcomes when compared to decompressive craniectomy performed >24 h. In fact, data from RCTs suggest that late decompressive craniectomy for TBI may result in worse functional outcomes than maximal medical therapy. In pediatric TBI patients, patients also had better functional outcomes when treated with decompressive craniectomy regardless of timing. High quality studies better informing the timing and indications for decompressive craniectomy are needed for both ischemic stroke and TBI. The additional data provided by imaging and advanced neuromonitoring could also be useful adjuncts in guiding decision making.

## Author Contributions

AS: Conceptualization of the manuscript, literature review, data analysis, and manuscript writing. SA: Data analysis and revision consultant. GH: Supervised, edited/wrote the manuscript and literature/data analysis.

### Conflict of Interest Statement

The authors declare that the research was conducted in the absence of any commercial or financial relationships that could be construed as a potential conflict of interest.
